# 

**Published:** 1910-01-29

**Authors:** 


### Gifts and Bequests.

The Morningfield Hospital, Aberdeen, has received a legacy of £25 from the late ex-Bailie George Walker.

Mrs. Jane Margaret Lewis, of the Holy Rood Convent, Worthing, has left £50 to the Brighton County Hospital.

The Court of the Carpenters' Company at their last meeting voted over £800 in donations and subscriptions to various charitable institutions.

The Clothworkers' Company has granted 50 guineas, and the Carpenters' Company 15 guineas to the Royal Hospital for Incurables, Putney Heath.

Miss Lydia Jane Cumming Rashleigh, of Abbey House, Netley, Southampton, has left £7,000 to be held upon trust for Margaret Jane Yates for life, with remainder to the Royal South Hants and Southampton Hospital, the income to be applied for the personal comfort, treatment, and good of the patients, and she also left her wines, spirits, and liquors to the hospital; and £500 to the Southampton Free Eye Hospital.

### Appointments.

Dr. Lechmere Anderson has been reappointed medical officer of health by the Doncaster Corporation.

Dr. Hawthorn has been appointed one of the medical officers to the Wellington Board of Guardians.

Dr. P. R. Keary, J.P., has been appointed medical officer of the Portumna (Ireland) No. 2 Dispensary District.

Dr. James Miller has been appointed medical officer of the burgh of Kirkintilloch in succession to the late Dr. William Whitelaw.

Dr. Andrew Norris Stevens has been appointed medical officer of health to the Yarmouth Corporation, in succession to Dr. Beach.

### THE QUESTION BOX.

**SPECIAL NOTICE.**—Questions of interest affecting the honorary medical staff and hospital administration in all departments are invited. When any point arises we hope our readers will formulate it in a question and so co-operate to make this column of real value and interest. In this way the experience of the best-managed hospitals should be made helpful to the whole hospital world. We shall welcome the contribution of both questions and answers.

N.B.—The publication of an answer in this column does not necessarily preclude the publication of subsequent answers to the same question, for a question may involve points which require to be threshed out and fully discussed. Answers, if published, will be paid for at scale rates.

### WOMEN ON HOSPITAL BOARDS.

Question: Is it desirable that women should be appointed as members of the governing and executive committees of voluntary hospitals?

### A Leicester View.

Mr. Harry Johnson, House Governor and Secretary of the Leicester Infirmary, writes:

Women have now for some years held seats on the Board of Governors and on the House Committee (the principal administrative committee). They have rendered very effective service in many directions, but have been specially helpful in connection with tenders for groceries and provision, bedding, linen, etc: In matters of detail in connection with the furnishing of the new nurses' home the lady members of the Board, with the lady superintendent, have helped to settle many points. The withdrawal of women representatives from the board and committees would be a very distinct loss to the institution. Women are also eligible for nomination as representative governors by bodies of workpeople, and the privilege has been taken advantage of to the gain of the Saturday hospital movement of the town.

### A Liverpool View.

By Mr. Francis H. Moore, General Superintendent and Secretary, Liverpool Royal Infirmary.

In neither of the two hospitals with which I have been associated in the management during the past thirty-four years —namely St. George's Hospital, Hyde Park Corner, and the Liverpool Royal Infirmary—have I had any experience of women as members of the governing body. I can testify to the admirable management of their departments of the various matrons with whom I have been in contact during that period—always with the utmost devotion and sympathy for the well-being of the women under their charges, and, with one exception, with a thorough desire to exercise their control with economy and for the general harmony of the household; and I cannot see that the presence of members of their sex (who must necessarily be less experienced in these matters) on a committee from which they hold their authority could in any way conduce to an improvement of these conditions, but quite the reverse.

### The View of a Hospital Secretary in the Midland

Women are now taking their share in the administrative work of most of our public institutions—*i.e.* Boards of Guardians, Education Authorities, etc.—and have demonstrated their usefulness on these bodies. Women on the boards of voluntary hospitals can be most helpful in many ways, and they should be encouraged to take an active part in their administration. An excellent plan of bringing this about is to form an Auxiliary Committee composed entirely of women. This committee should be given adequate representation on the General Committee; they should have referred to them certain matters for which help in the choosing of the half-yearly tenders for supplies, also linen tenders—they are more acquainted with these household necessities than men. It is impossible to do without their aid when running bazaars, and men will not undertake house-to-house collections; the ladies will, and from experience I can guarantee that a steadily increasing annual income can be obtained from this source when thoroughly organised with an influential Secretary at the head. Women are more suited to visit and cheer the female patients, and if a convalescent home is attached, they are excellent as visitors. With such a committee, Sewing Guilds could also be formed, for the purpose of making articles of clothing for the patients' comfort.

### A POINT FOR COTTAGE HOSPITAL ADMINISTRATORS.

Question: Is it usual or advisable for the Matron of a Cottage Hospital to have the right to make use of a private ward for her private guests?

### HOSPITAL PROBLEMS WHICH PRESS.

### THE RIGHT TO PAY FOR HOSPITAL TREATMENT.

The second article on this subject is unavoidably held over till next week.

### THE GRADUATES' MEDICAL SCHOOLS.

DIARY FOR THE WEEK, JAN. 31 to FEB. 5.

**CENTRAL LONDON THROAT AND EAR HOSPITAL, Gray's Inn Road, W.C.**

**At 3.45 p.m.—Feb. 1,** Dr. Atkinson, **Nose.**
**4.30 p.m.—Feb. 3,** Dr. Atkinson, **Syphilitis of the Throat.**
**3.45 p.m.—Feb. 4,** Dr. Dan McKenzie, **Labyrinth.**

**THE BROMPTON HOSPITAL FOR CONSUMPTION AND DISEASES OF THE CHEST, Fulham Road, S.W.**

**At 4 p.m.—Feb. 2,** Dr. Habershon, **The Early Signs of Pulmonary Tuberculosis.**

**ST. JOHN'S HOSPITAL FOR DISEASES OF THE SKIN, Leicester Square, W.C.**

**At 6 p.m.—Feb. 3,** Chesterfield Lecture: **Pityriasis Rubra Pilaris and Lichen, and the Treatment of each.**

**LONDON SCHOOL OF CLINICAL MEDICINE, Seamen's Hospital, Greenwich, S.E.**

**At 3.30 p.m.—Feb. 2,** Mr. Cargill, **The Normal Fundus Oculi and its Common Anomalies.**
**2.30 p.m.—Feb. 3,** Dr. Rankin, **Meningitis and its Treatment.**
**3.15 p.m.—Feb. 4,** Mr. McGavin, **Operative and Post-operative Accidents.**

**MEDICAL GRADUATES' COLLEGE AND POLYCLINIC, 22 Chenies Street, W.C.**

**At 4 p.m.—Jan. 31,** Dr. James Galloway, **Clinique (Skin).**
**5.15 p.m.—Jan. 31,** Mr. Nathan Raw (Liverpool), **Treatment of the Surgical Forms of Tuberculosis by Koch's Human Tuberculin.**
**4 p.m.—Feb. 1,** Dr. Walter Carr, **Clinique (Medical).**
**5.15 p.m.—Feb. 1,** Mr. H. L. Eason, **Affections of the Eye caused by Inflammation of the Accessory Sinuses of the Nose.**
**4 p.m.—Feb. 2,** Mr. James Berry, **Clinique (Surgical).**
**5.15 p.m.—Feb. 2,** Dr. Fitzgerald Powell, **The Treatment of Mastoiditis.**
**4 p.m.—Feb. 3,** Sir Jonathan Hutchinson, **Clinique (Surgical).**
**5.15 p.m.—Feb. 3,** Mr. Jackson Clarke, **Some Painful Surgical Conditions of the Feet.**
**4 p.m.—Feb. 4,** Mr. Herbert Tilley, **Clinique (Ear, Nose, and Throat).**

**THE POST-GRADUATE COLLEGE, West London Hospital, Hammersmith, W.**

**At 10 a.m.—Jan. 31 and Feb. 3, Demonstration of Cases by Surgical Registrar.**
**10 a.m.—Feb. 4, Demonstration of Cases by Medical Registrar.**
**12 noon.—Feb. 3,** Dr. Bernstein, **Pathological Demonstration.**
**12.15 p.m.—Feb. 1,** Dr. Pritchard, **Practical Medicine.**
**12.15 p.m.—Feb. 2,** Dr. Grainger Stewart, **Practical Medicine.**
**5 p.m.—Jan. 31,** Mr. Baldwin, **Practical Surgery.**
**5 p.m.—Feb. 1,** Dr. Robinson, **Gynæcology.**
**5 p.m.—Feb. 2,** Dr. Beddard, **Medicine.**
**5 p.m.—Feb. 3,** Dr. Grainger Stewart, **The Significance of Ocular Paralysis in the Diagnosis of Nervous Disease.**
**5 p.m.—Feb. 4,** Dr. Low, **Sleeping Sickness.**

**THE HOSPITAL FOR SICK CHILDREN, Great Ormond Street, W.C.**

**At 4 p.m.—Feb. 3,** Mr. Waugh, **Spinal Caries.**

**NORTH-EAST LONDON POST-GRADUATE COLLEGE, Prince of Wales's General Hospital, Tottenham, N.**

**At 4.30 p.m.—Feb. 1,** Mr. Carson, **Intussusception.**

**ROYAL SOCIETY OF MEDICINE, 20 Hanover Square, W.**

**At 4.30 p.m.—Feb. 1, Therapeutical and Pharmacological Section:**

*Paper.*—Dr. Bashford: "The immunity reaction of cancer."

A discussion on "Proprietary, patent, and secret remedies" will be opened by Professor W. E. Dixon.

(A meeting of the Council of the Section will be held at 4 p.m.)

**At 8.30 p.m.—Pathological Section:**

*At the National Hospital, Queen Square, W.C.*

*Cases.*—Dr. F. W. Mott, F.R.S., and Dr. Edgar Silvester: Changes in the nerve-cells of a man who died seven hours after receiving a current of 10,000 volts. Dr. F. W. Mott, F.R.S.: Two specimens from Professor Marinesco illustrating regeneration of nerves. Dr. A. J. Hall: Two cases of cyst of choroid plexus. Dr. Batten and Dr. Gordon Holmes: A recent case of acute poliomyelitis. Dr. Hinds Howell: Two cases of tumour of the pineal gland. And other communications.

**At 5 p.m.—Feb. 4, Laryngological Section:**

*Cases and Specimens.*—Dr. H. J. Davis and Dr. G. Wilkinson.

**At 8.30 p.m.—Section of Anæsthetics:**

An adjourned discussion on Mr. Bellamy Gardner's paper on "Lymphatism" will be opened by Dr. Farquhar Buzzard.

**At 10 a.m.—Feb. 5, Otological Section:**

*Cases and Specimens.*

### APPOINTMENTS VACANT.

ST. JOHN'S HOSPITAL FOR DISEASES OF THE SKIN.—Pathologist.

